# Mucosal Leishmaniasis Involving the Nostril and Maxillary Sinus: A Case Report

**DOI:** 10.7759/cureus.30289

**Published:** 2022-10-14

**Authors:** Rahul R Bhowate, Pragati A Bhargava, Simran D Badki, Mrunal Meshram

**Affiliations:** 1 Department of Oral Medicine and Radiology, Sharad Pawar Dental College and Hospital, Datta Meghe Institute of Medical Sciences (Deemed to be University), Wardha, IND

**Keywords:** rhinorrhea, donovan bodies, bitot's spot, polypoid nasal swelling, nasal stuffiness

## Abstract

An eight-year-old child presented with nasal discharge, stuffiness, and whitish polypoid swelling in the left nostril with Bitot’s spot. Computed tomography (CT) evaluation showed complete involvement of the left nostril and maxillary sinus. Blood investigations revealed leukocytosis, raised absolute eosinophils, increased alkaline phosphatase, and reduced vitamin A levels. Histopathological examination revealed inflammatory infiltrate with Leishman-Donovan bodies, which confirms the diagnosis of mucosal leishmaniasis (ML). In the present case, the recording of demographic data is important as the child was a migrant from the leishmaniasis-endemic area of Bihar state, India. Nasal polypoid growth was removed by endoscopic surgery, followed by a combination of allopathic and polyherbal preparation. The child responded well to these therapeutic measures, and there was no recurrence of nasal discharge, stuffiness, and crustation at six-month follow-up.

## Introduction

*Leishmania* parasite mainly resides in the macrophages, transmitted by female sand flies to host tissue. Host immune response and *Leishmania* species are responsible for local or systemic inflammation involving the reticuloendothelial system. Broadly, leishmaniasis is classified into visceral and tegumentary. Visceral leishmaniasis (VL or kala-azar) is responsible for dreaded systemic conditions presenting with fever, weight loss, asthenia, hepatosplenomegaly, and pancytopenia. In visceral leishmaniasis, 90% of the global burden is reported from India, Sudan, Somalia, Brazil, and Ethiopia [1,2]. In India, more than 50% of cases (75% of the total global cases) are reported from Bihar state, and 61.1% account for visceral leishmaniasis [3,4]. Cutaneous leishmaniasis (CL) has been reported in a non-endemic area [5,6]. In Kerala, Punjab, and Rajasthan, CL cases are mainly caused by *Leishmania tropica*, and in Himachal Pradesh, *Leishmania donovani* and *L. tropica* are responsible for CL [7,8]. Facial, arm, and leg cutaneous lesions are common in systemic leishmaniasis [9,10]. Tegumentary leishmaniasis commonly involves cutaneous and mucosal areas and may cause localized or diffuse lesions [11]. Scraping from cutaneous and mucocutaneous lesions demonstrated that amastigotes (Leishman-Donovan bodies) are a simple and easy method of diagnosis and show positive results in 50.70% of the cases [6,12]. Directorate General of Health Services (DGHS, New Delhi) reports revealed that the elimination target for systemic leishmaniasis and the endemic district of Bihar was achieved in Mahua Block; i.e., in 2012, the case incidence rate was 4.5 per 10000 people and 0.5 in 2016 [4]. The present case was a migrant from Mahua Block of Patna reported with the delayed eruption of maxillary anterior teeth, nasal discharge, difficulty in breathing, and stuffiness of the left nostril and reported as mucosal leishmaniasis (ML) on histopathological examination.

## Case presentation

An eight-year-old male reported delayed eruption of maxillary anterior teeth, nasal discharge, and intermittent fever for the last two years. Difficulty in breathing and stuffiness of the left nostril were present in the last one year. The patient’s father was a migrant worker in Maharashtra from Mahua, Patna district, in the state of Bihar, India. The child was residing in Mahua along with their grandparents and doing his schooling. The grandparent’s occupation was crop and dairy farming. The parents stated that the child was suffering from intermittent fever in the afternoon and evening hours and continuous nasal discharge from the left nostril for the last two years. At Mahua, the child received treatment from a local physician without any relief. For the last one year, the child was suffering from stuffiness in the left nostril and difficulty in breathing. For the last three months, the parents brought the child for treatment at their workplace in Maharashtra, and the child was treated by the local physician but not relieved from stuffiness and breathing difficulties; therefore, the concerned physician referred this case to our hospital.

**Figure 1:**
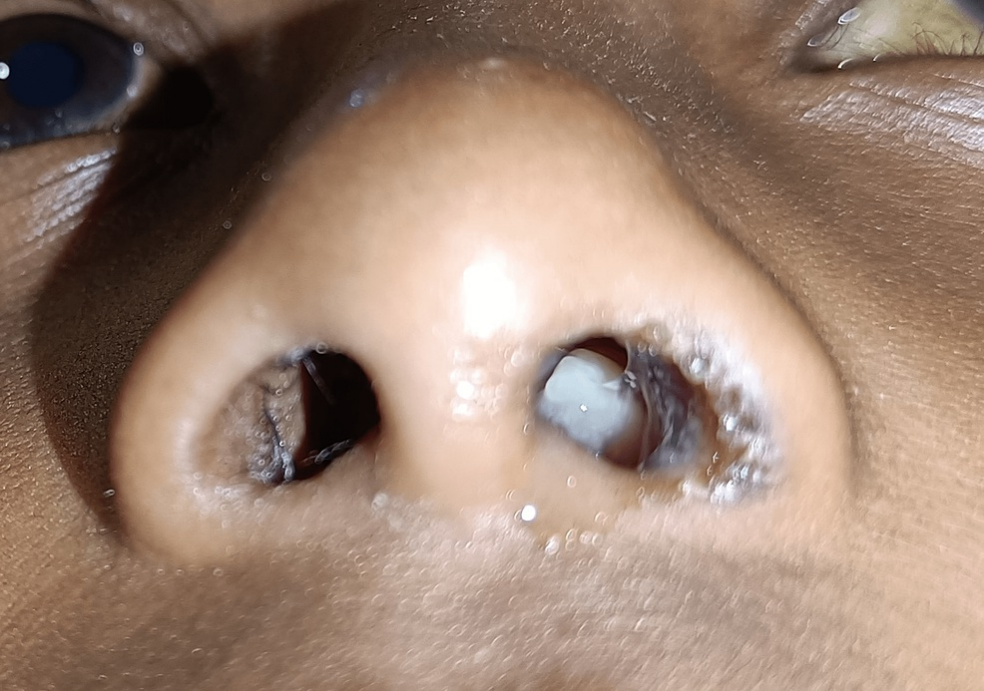
Whitish polypoid lesion in the left nostril with crustation on the left mucocutaneous region of the nose

**Figure 2:**
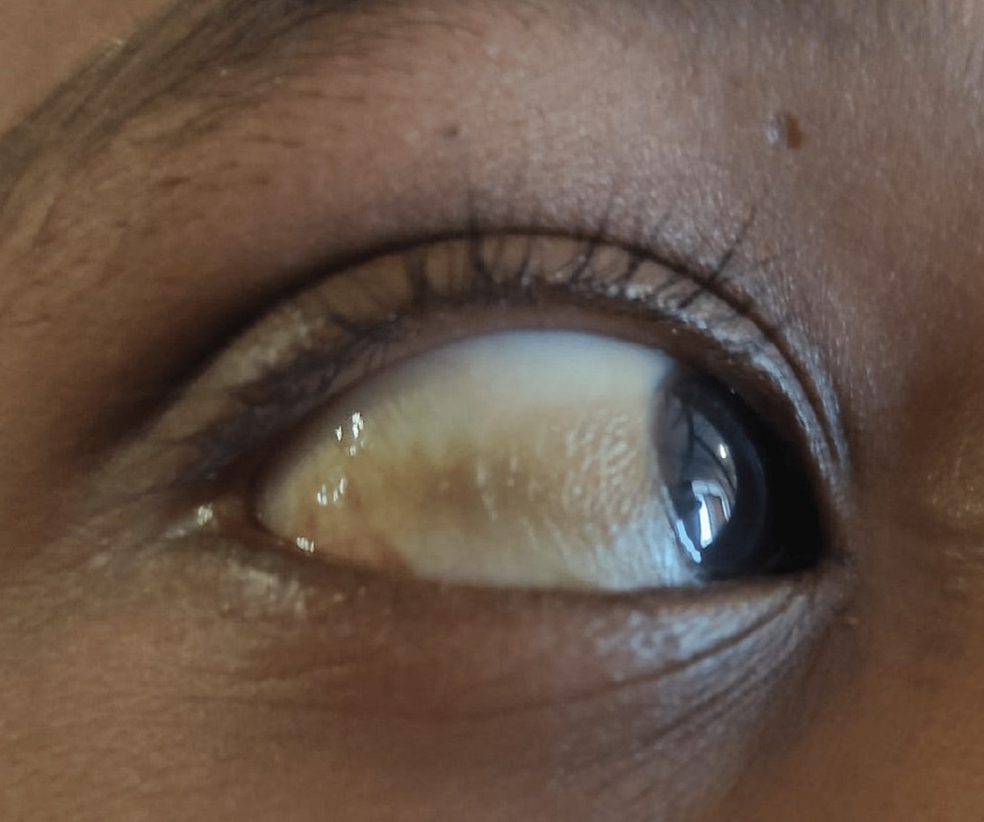
Bitot’s spot Bitot’s spot is a triangular patch on the conjunctiva

Extra-oral examination revealed asymmetric diffuse facial swelling on the left midfacial region. The left nostril showed a whitish polypoid lesion obstructing the air passage with mucosal crustation of the lateral wall of the nasal cavity and at the mucocutaneous junction with congested right nostril mucosa (Figure 1) and Bitot’s spot (Figure 2), suggestive of vitamin A deficiency. Intra-oral examination revealed the non-eruption of maxillary anterior teeth.

**Figure 3:**
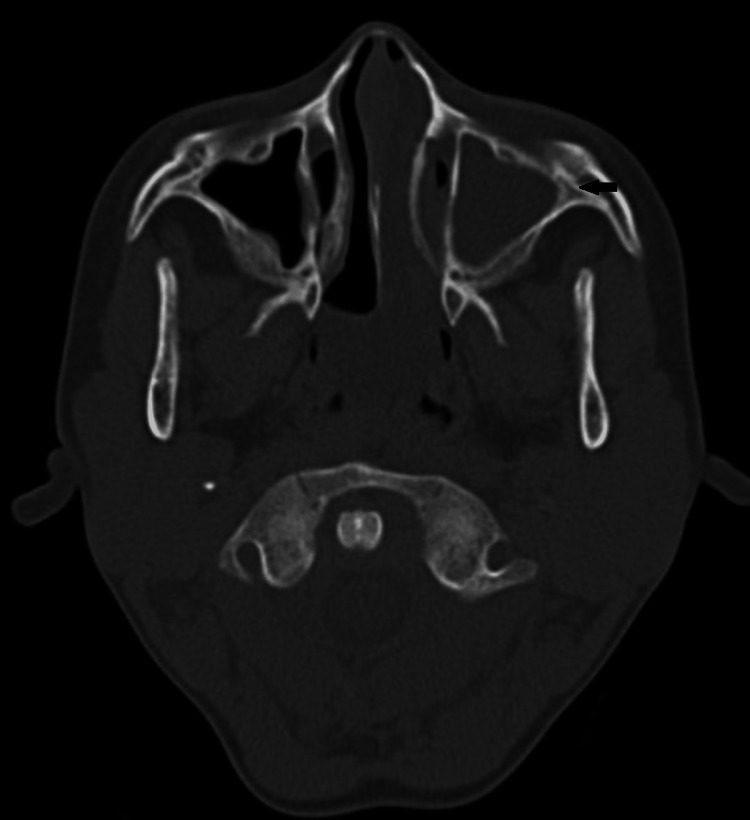
Axial CT scan showing complete haziness of the left maxillary sinus and nostril with thinning of the nasal septum CT: computed tomography

**Figure 4:**
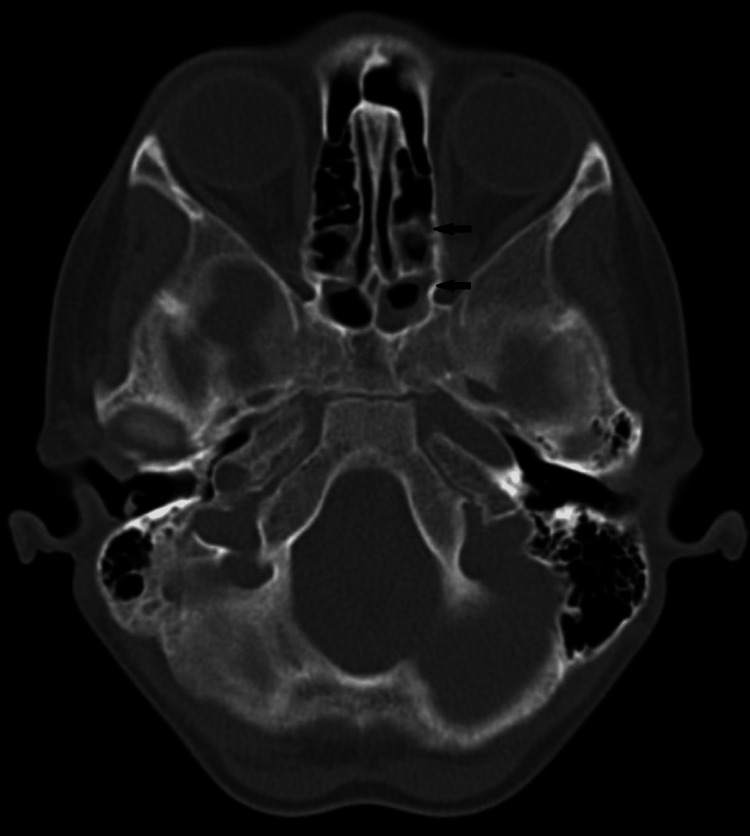
Axial CT scan showing partial mucosal thickening of ethmoid and sphenoid sinuses (black arrow) CT: computed tomography

Computed tomography (CT) scan examination of the maxillary sinus revealed loss of nasal septum and reduced medial wall certification of the left maxillary antrum with complete haziness (Figure 3) and partial mucosal thickening with ethmoid and sphenoid sinuses (Figure 4). Considering the clinical course of the disease, clinical presentation, CT scan evaluation, and demographic details of the patient favor clinical and imaging diagnosis of tegumentary mucosal leishmaniasis involving the left nostril and maxillary sinus. A cytological smear taken from the nasal mucosa showed plenty of chronic inflammatory cells.

Blood investigations showed leukocyte count of 14100 cells/cumm, increased absolute eosinophil count of 1128 cells/cumm, decreased vitamin A level of 0.88 mmol/L, and increased alkaline phosphates of 438 (Table 1).

**Table 1:** Blood investigations RBC: red blood cell (4.3-5.7 millions/cumm); MCH: mean corpuscular hemoglobin concentration (33%-37%); MCV: mean corpuscular volume (80-100 fl); MCH: mean corpuscular hemoglobin (26-34 pg); HCT: hematocrit (41%-50%); Hb: hemoglobin (13.5-17.5 g/dl); RDW: red cell distribution width; WBC: white blood cells (4.5-11.0 cells/cumm); AEC: absolute eosinophils count (>500 cells/mcL); APTT: activated partial thromboplastin clotting time (20-40 seconds); PT: prothrombin time (10-14 seconds); INR: international normalized ratio (below 1.1); ALT: alanine transaminase (0-40 U/L); AST: aspartate aminotransferase (7-40 U/L)

Investigation	Value
RBC	4.45 millions/cumm
MCHC	33.9%
MCV	80.6 fl
MCH	27.3 pg
HCT	35.9%
Hb	12.2 g/dl
RDW	14.6%
WBC	14100 cells/cumm
Monocyte	4%
Granulocyte	50%
Lymphocyte	38%
Basophils	0%
Eosinophils	8%
AEC	1128 cells/mcL
Platelet count	2.97 lacs/cumm
APTT	32.0 seconds
PT	14.3 seconds
INR	1.27
Serum ferritin	19.4 ug/L
Serum iron	149 mcg/dl
Serum urea	25 mg/dl
Creatinine	0.5 mg/dl
ALT	18 U/L
AST	26 U/L
Alkaline phosphatase	438 U/L
Conjugated bilirubin	0.1 mg/dl
Unconjugated bilirubin	0.2 mg/dl
Vitamin A	0.88 mmol/L

**Figure 5:**
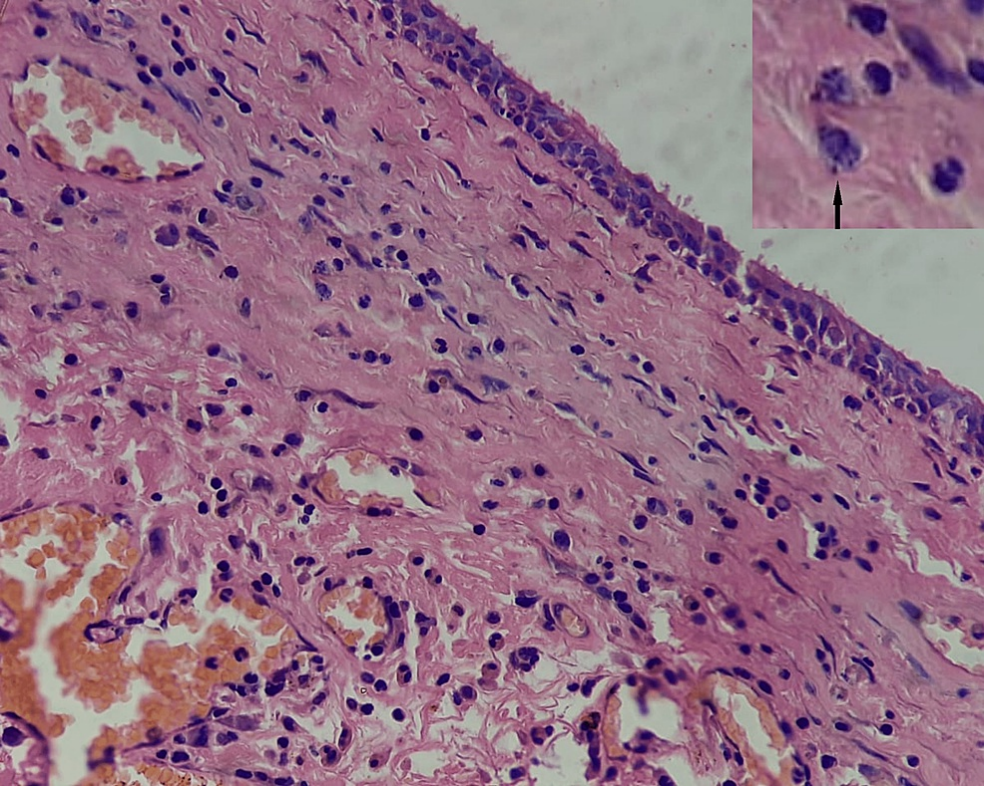
Photomicrograph (40×) showing chronic inflammatory cell infiltrate and inset image showing Leishman-Donovan bodies (black arrow)

Endoscopic biopsy from nasal polypoid tissue on histopathological examination showed chronic inflammatory cells, lymphocytes, and histiocytes, and the cytoplasm of macrophages showed *Leishmania* amastigote (Figure 5). Nasal polypoid growth was completely removed by endoscopic surgery under general anesthesia. Before surgical intervention, the combination of allopathic and herbal preparations was started. The patient was started with albendazole syrup 400 mg single dose, vitamin A 1500 IU per day twice daily for months, itraconazole 100 mg twice daily at mealtimes for one day, silymarin 70 mg daily for two months, and Haridrakhand 5 g twice daily prepared with fresh decoction with lukewarm water for two months. After one month, white blood cell (WBC) count, absolute eosinophil count, liver function test (LFT), kidney function test (KFT), and vitamin A were within normal levels.

**Figure 6:**
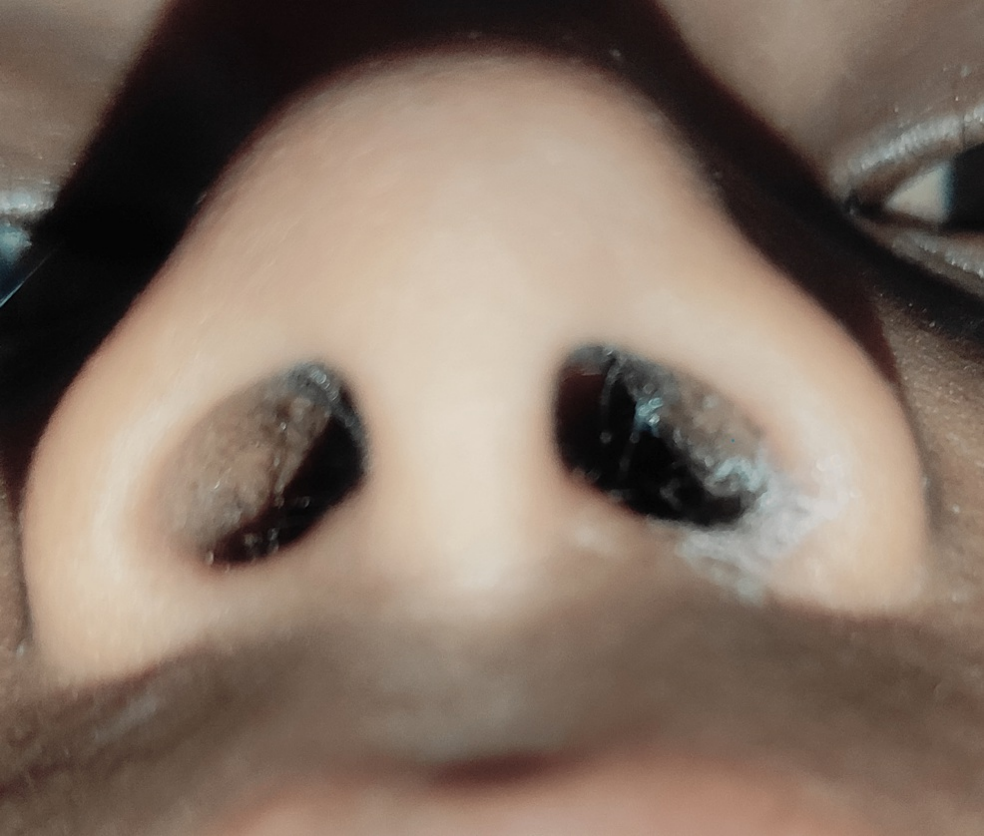
Post-treatment clinical photograph showing clear nasal passage in the left nostril

**Figure 7:**
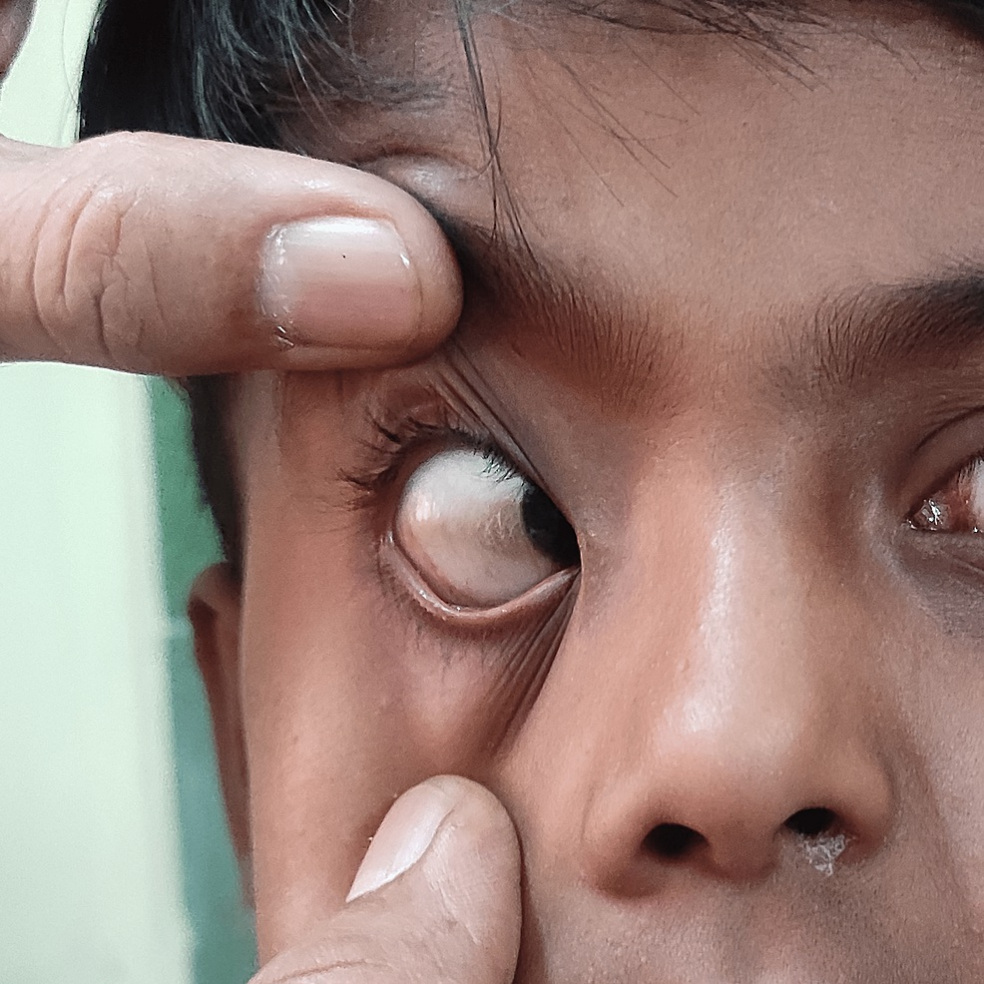
Improved Bitot's spot Post-treatment clinical photograph shows clearing triangular patch of conjunctiva

Post-treatment healing was uneventful, and six-month follow-up did not reveal any nasal discharge, stuffiness of nostrils or difficulty in breathing, and nasal crustation (Figures 6-7).

## Discussion

Mucosal leishmaniasis (ML) is a clinical presentation of asymptomatic CL or the individuals that recovered from CL, which usually takes 1-2-year period [13]. From the recovered CL cases, 3%-5% develops into ML [14,15]. Molecular blood analysis has an equal potential for diagnosis in CL and ML and showed better sensitivity than bone marrow or spleen aspirates [16,17]. But parasitic DNA detection in blood samples before the clinical presentation of ML [18] and blood culture for parasites help in the diagnosis of ML cases [19]. Old-world leishmaniasis is limited to the skin, and systemic leishmaniasis spreads through the vascular system and involves vital organs. Clinical presentations in new-world leishmaniasis are cutaneous or mucosal lesion, which involves mucosal tissues of the upper respiratory tract and oral cavity [20]. ML is preceded by cutaneous or systemic leishmaniasis, and rare initial mucosal involvement is caused by *Leishmania donovani* complex species, commonly *L. infantum*, in the Indian population [21]. Local mucosal immunodeficiency due to tobacco use, gingival and periodontal diseases, a local or systemic corticosteroid drug, altered oral microbial flora, and upper respiratory diseases may increase the chances of ML in the endemic region [22].

Mucosal spread from either CL or VL occurs in the following manner: (i) ML is spread from the adjacent cutaneous lesion by continuity and contiguity caused by *L. major* [23]; (ii) ML from *Leishmania braziliensis* is complex preceded by healed skin lesions of the arms, trunk, and legs and spread through hematogenous/lymphatic routes to mucosal sites by *L. infantum* [22,24], and in isolated ML, it is difficult to identify whether the reactivation of latent parasites, a new infection [6], or altered local immune response of the mucosa in immunocompetent patients is responsible for lesion [24]; and (iii) sand fly bite may be possible for oral and nasal mucosal lesions [25,26]. Initially, ML affects the sinonasal area, oral mucosa, and upper respiratory tract and later on involves the laryngeal and pharyngeal areas as the disease progresses. Sinonasal involvement in ML cases presented with stuffiness of the nose, inflammation, edema, serous rhinorrhea, and epistaxis that may progress to dysphagia and dysphonia. Diffuse mucosal involvement of the oropharyngeal and sinonasal areas may easily spread to the lower respiratory tract [20,22,24,26,27].

Systemic leishmaniasis caused by *L. donovani* and *L. infantum* is responsible for ML in immunocompromised patients, and isolated ML patients were reported to involve the mucosa of the nostril, oropharynx, and larynx, due to unchanged local immune response [22]. Oral ML caused by *L. donovani* induces tooth loss and breathing difficulty as the severity increases. The nasal mucosa is presented with whitish polypoid growth in the background of inflammatory mucosa with frustrations at the mucocutaneous region [28]. In the present case, the child presented with nasal discharge for two years and difficulty in breathing for one year, and clinical examination revealed whitish polypoid swelling in the background of the inflammatory nasal mucosa of the left nostril with peripheral crustation without any cutaneous lesions on facial or upper or lower limb region. Histopathological examination revealed *Leishmania* amastigote in the mucosal lesions on Giemsa and hematoxylin and eosin (H&E) staining [27].

de Camargo et al. reported various structural changes in CT scans of treated ML cases. The erosion of the nasal septum and the nasal bone, the thickening of the nasal mucosa, and the haziness of the maxillary sinus with loss of certification involving the nasopharynx and soft palate were reported as a characteristic destructive feature in ML [29]. Similarly, histopathological finding with abundant inflammatory cell infiltration with intracellular Leishman-Donovan body was present in this case. Also, CT imaging evaluation showed complete involvement of the left nasal cavity and maxillary sinus on the left side with partial thickening of ethmoid and sphenoid sinuses. Malnutrition is a risk factor for the development of systemic leishmaniasis [30]. Kala-azar patients reported low serum levels of vitamin A and zinc [31]. Improved clinical outcome of the disease was reported in children with kala-azar who received zinc supplementation along with amphotericin B or pentavalent antimonial treatment [32,33]. The serum levels of vitamin A, vitamin D, zinc, and iron improve the treatment outcomes in systemic leishmaniasis [34]. In the present case, the child also suffered from Bitot’s spot, suggestive of vitamin A deficiency.

Fouladvand et al. (2013) [35], Chouhan et al. (2015) [36], and Afrin et al. (2019) [37] reported the anti-leishmaniasis property of curcumin, *Piper nigrum*, and *Cinnamomum cassia* against *Leishmania donovani* promastigotes and amastigotes and proved inhibitory effects in vivo without hepatic and renal toxicity. Considering this evidence, the child was started with Haridrakhand, which is a polyherbal preparation containing turmeric, cinnamon, and piperine including another active ingredient of Indian herbs. Turmeric, cinnamon, and piperine may act in synergism and are responsible for favorable effects in this neglected parasitic infection. Similarly, Galvão et al. (2017) [38] concluded in a systematic review that itraconazole was effective in mucosal leishmaniasis because of its optimum bioavailability and it maintains higher tissue concentrations compared to the other azoles, including in the mucosa. Therefore, the child was started with itraconazole. Considering the hepatotoxicity of itraconazole, the patient was started with silymarin, which also has synergism with turmeric and fortified the hepatoprotective activity [39].

## Conclusions

In endemic areas, migrant individuals from the endemic area of leishmaniasis should be considered as a differential for nasal obstructive masses. Although mucosal leishmaniasis is more uncommon in our region than the VL or post-kala-azar dermal leishmaniasis (PKDL), patients with facial cutaneous lesions are required to be evaluated for oral, mucosal, sinonasal, and oropharyngeal lesions.

In the present case, the child presented with a chronic obstructive mass in the left nostril for the last one year, which on CT evaluation revealed rhinosinusitis. Also, the child was suffering from Bitot’s spot, indicative of vitamin A deficiency, being a resident of an endemic area for leishmaniasis. These correlations contribute to the diagnosis of mucosal leishmaniasis for early initiation of treatment. Considering the evidence-based effectiveness of the Indian spices and herbs on this parasitic infection, the present case was considered for polyherbal preparation with allopathic medication for patient benefit.
